# An Evidenced-Based Review of the Prevention of Venous Thromboembolism in Traumatic Patients with Intermittent Pneumatic Compression Devices

**DOI:** 10.1155/2023/2116496

**Published:** 2023-08-07

**Authors:** Meng Zeng, Zhoupeng Wu

**Affiliations:** ^1^Department of Orthopedics, West China Hospital, Sichuan University, 37 GuoXue Alley, Chengdu 610041, Sichuan, China; ^2^Department of Vascular Surgery, West China Hospital, Sichuan University, 37 GuoXue Alley, Chengdu 610041, Sichuan, China

## Abstract

**Objective:**

To search, evaluate, and summarize the best evidence of intermittent pneumatic compression devices to prevent venous thromboembolism in trauma patients.

**Method:**

Evidence retrieval was conducted from top to bottom according to the “6*S*” evidence model, including guidelines, clinical decision making, evidence summary, expert consensus, and systematic reviews. The retrieval time limit was from the establishment of the database to August 31, 2022. Two researchers independently evaluated the quality of the literature, extracted evidence, and summarized evidence.

**Results:**

A total of 140 studies were obtained in the literature retrieval, and 50 studies were obtained after rechecking and reading the title and abstract. After combining the inclusion and exclusion criteria, 19 studies were finally included. Among them, there were 12 guidelines, 1 clinical decision making, 1 evidence summary, 1 expert consensus, and 4 systematic reviews. The 27 best pieces of evidence were summarized from the four dimensions of pretreatment evaluation, contraindications and applicable conditions, treatment strategies, training, and patient education.

**Conclusion:**

This study summarized the evidence of using an intermittent pneumatic compression device to prevent venous thromboembolism in trauma patients and provided the basis for scientific and effective standardized management of mechanical thromboembolism prevention. When applying evidence, it is necessary to combine clinical practice and patient wishes and select evidence pertinent to improving the effectiveness of intermittent pneumatic compression devices in preventing venous thromboembolism. In addition, compliance with the clinical application of IPC is relatively low, so we should start from two aspects before the application of evidence. We should not only increase the number of IPCs but also strengthen the training of VTE prevention knowledge and practical skills of medical staff to provide good health education for patients and their families to improve compliance with the clinical application of IPC.

## 1. Introduction

Trauma has become one of the main problems affecting health [1], and its mortality rate ranks third in the disease mortality spectrum [2]. Venous thromboembolism (VTE), including deep venous thrombosis (DVT) and pulmonary embolism (PE) [3], is a common complication in trauma patients [4] and the main cause of death in hospitalized trauma patients [5]. The risk of hospital-related VTE after multiple traumas is high, at more than 50% [6, 7]. Therefore, as a high-risk group for VTE, it is necessary for trauma patients to carry out early and active thrombosis prevention [5, 8]. Mechanical thromboprophylaxis is widely used in trauma patients because of its good practicability, simple operation, few complications, and low bleeding risk [4, 5]. When there are contraindications to using drugs to prevent VTE, patients with trauma will implement mechanical thrombosis prevention [9]. The guide also recommends intermittent pneumatic compression devices (IPCs) for the prevention of VTE in trauma patients [10, 11]. IPC is a kind of circulatory inflation through a compression device to simulate the contraction and relaxation of muscle movement to strengthen the blood flow of the deep vein and prevent thrombus [9, 12]. At present, IPC mechanical prevention is often used as a supplement to drug prevention. Although many guidelines on the treatment and management of VTE have been issued internationally, many hospitals have formulated various strategies to prevent VTE, but the formulation of preventive measures lacks a certain evidence-based basis. This study uses evidence-based research methods to comprehensively summarize the best evidence of using IPC to prevent VTE in trauma patients, aiming to provide operable reference standards for the implementation of scientific and effective mechanical thrombosis prevention.

## 2. Methods

### 2.1. Clarifying Research Issues

We used the PIPOST model developed by JBI as a tool to construct evidence-based questions [13], that is, the target population of evidence application *P* (population): trauma patients, age ≥18 years old; Intervention *I*: IPC; *P* (professional): hospital managers and medical staff; Outcome index *O* (outcome): DVT incidence, pulmonary embolism incidence, VTE incidence; Evidence application site *S* (setting): hospital (surgery, trauma services, ICU); Type of evidence *T* (type of evidence): guidelines, systematic reviews, evidence summary, clinical decision making, expert consensus/opinion/position statement, and original research closely related to evidence.

### 2.2. Literature Retrieval Strategy

We used “trauma^*∗*^” OR “polytrauma” OR “wound^*∗*^” OR “injury^*∗*^” OR “multiple injury”) AND (“intermittent pneumatic compression devices^*∗*^” OR “intermittent pneumatic compression^*∗*^” OR “pneumatic compression^*∗*^” OR “sequential compression devices^*∗*^” OR “pneumatic compression hose” OR “mechanical thromboprophylaxis”) AND (“venous thromboembolism^*∗*^” OR “deep vein thrombosis^*∗*^” OR “Thromboembolism^*∗*^” OR “thromboprophylaxis^*∗*^” as the search keywords. According to the “6*S*” evidence model, we searched BMJ Best Practice (1), UpToDate (4), Joanna Briggs Institute Evidence-Based Health Care Center Database (JBI) (1), National Guidelines Clearinghouse (NGC) (1), National Institute for Health and Care Excellence (NICE) Registered Nurses' Association of Ontario (RNAO) (0), Scottish Intercollegiate Guidelines Network (SIGN) (1), Guidelines International Network (GIN) (1), EBSCO (0), Cochrane Library (1), PubMed (9), Embase (0), Cumulative Index to Nursing and Allied Health Literature (CINAHL) (0), American College of Chest Physicians (ACCP) (0), and European Society for Vascular Surgery (ESVS) (0). The retrieval time was from the establishment of the database to August 31, 2022. Literature inclusion criteria were as follows: the subjects were trauma inpatients aged ≥18 years old; the content of the literature involved the evaluation, intervention, or management of VTE prevention by using intermittent air pressure therapy devices in trauma patients; and evidence types included guidelines, clinical decision making, evidence summary, expert consensus, systematic reviews, and original research related to evidence. Exclusion criteria were as follows: (1) documents with duplicate publications, incomplete information, and inability to obtain full text; (2) document types mainly include plans, drafts, reports, and abstracts; and (3) research that did not pass document quality evaluation.

### 2.3. Literature Quality Evaluation

The quality evaluation of the guidelines adopts the 2012 version of the Appraisal of Guidelines for Research and Evaluation (AGREE II) [14]. The scale covers a total of 23 items in 6 fields and 2 additional overall evaluation items. Each item is evaluated from 1 point (strongly disagree) to 7 points (strongly agree). The score of each field is equal to the total score of the items included in the field and is standardized as the percentage of the highest possible score in the field. The additional 2 overall evaluation items are scored from 1 to 7 points (1 = lowest quality; 7 = highest quality). The AMSTER (Assessment of Multiple Systematic Reviews) scale is used for systematic reviews [15]. The scale includes 11 items, and the item evaluation options can be “Yes, No, unclear, and not used.” The quality evaluation standard of expert consensus adopts the expert consensus evaluation standard of Australia's JBI Evidence-Based Health Care Center [16] to evaluate such research. The evaluation tool includes 7 items, and each item is judged by “Yes, No, unclear, and inapplicable.” Evidence summary and clinical decision making are based on the original evidence level and recommendation level.

### 2.4. Evidence Classification and Recommendation Level

**Figure 1:**
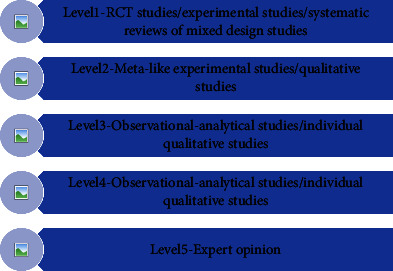
JBI grading of evidence for the study.

**Figure 2:**
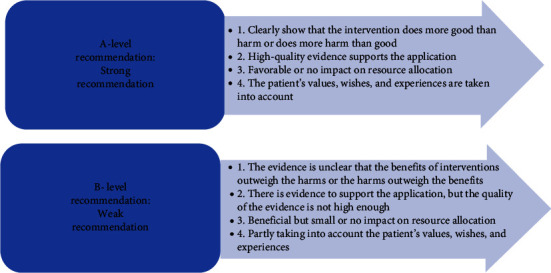
JBI recommended level of evidence for research.

An evidence-based group consisting of 10 experts in the field of critical care medicine and traumatology was established, including 2 evidence-based experts, 2 doctors in the department of critical care medicine, 2 doctors in the trauma services, 2 vascular surgeons, 1 nurse in the department of critical care medicine, and 1 nurse in the trauma services. The level and recommendation level of evidence are determined by the JBI evidence preclassification and evidence recommendation level system (2014 version) [17]. According to different types of evidence, the system divides them into Levels 1∼5 (Level 1 is the highest level, and Level 5 is the lowest level). The level of evidence is related to the rigor of the research design. At the same time, the recommendation strength of the selected evidence is determined according to its FAME attributes (validity, feasibility, suitability, and clinical significance), which are level *A* recommendation (strong recommendation) and level *B* recommendation (weak recommendation) (Figures 1 and 2).

### 2.5. Literature Quality Evaluation Process

Two researchers who have received evidence-based medicine training will conduct independent evaluations according to the corresponding standards of different document types. If the evaluation results cannot be agreed upon, the third evidence-based medicine expert can discuss until reaching a consensus. When the conclusions of evidence from different sources are in conflict, the inclusion principle followed in this paper is evidence-based evidence first, high-quality evidence first, and the latest published literature first.

## 3. Results

### 3.1. Literature Retrieval Results

A total of 140 studies were obtained in the literature retrieval, and 50 studies were obtained after rechecking and reading the title and abstract. After combining the inclusion and exclusion criteria, 19 studies were finally included. Among them, there were 12 guidelines, 1 clinical decision making, 1 evidence summary, 1 expert consensus, and 4 systematic reviews (see Table 1 for the general characteristics of the included studies).

### 3.2. Literature Quality Evaluation Results

#### 3.2.1. Quality Evaluation of the Guide

In this study, the correlation coefficients within different groups was used to test the consistency of the quality evaluation of the guidelines. A total of 12 guidelines [6, 10, 11, 18–26] were included in this study. The ICC values were >0.8, and the consistency of the evaluation was good.  Table 2 shows the standardized percentage and comprehensive evaluation results of each field of the guide.

#### 3.2.2. Quality Evaluation of Clinical Decision Making and Evidence Summary

This study included 1 clinical decision from UpToDate [5] and 1 summary of evidence from JBI [27]. A total of 6 studies were cited, all of which were based on the existing evidence level and recommendation level of the original text.

#### 3.2.3. Quality Evaluation of Expert Consensus

This study included one expert consensus. The reference [28]is from the United States Trauma Surgery Intensive Care Board. In this expert consensus, according to item 6, ‘Is there any inconsistency between the proposed points and previous research', is evaluated as ‘No', and the other five items are evaluated as ‘Yes'. The overall quality of this expert consensus is high.

#### 3.2.4. Quality Evaluation of Systematic Reviews

This study included four systematic reviews [29–32], one from the Cochrane Library and three from PubMed. Among them, the evaluation results of 11 items in the study of Lozano et al. [29] are “yes,” with high overall quality, and are allowed to be included. In O'Connell et al.'s study, according to the fourth item “has the publication been included in the inclusion criteria”, its evaluation result was “no”, while the evaluation results of other items were “yes”. Overall, this study has a complete design, high overall quality, and can be used as an included literature.

### 3.3. Summary of Evidence

This study systematically evaluated and analyzed 19 studies, comprehensively sorted out the evidence, and finally formed four dimensions of pretreatment evaluation, taboos and applicable conditions, treatment strategies, training, and patient education, with 27 best pieces of evidence (Table 3).

## 4. Discussion

### 4.1. Pretreatment Evaluation

Trauma patients are high-risk patients with VTE. All hospitalized trauma patients should undergo VTE risk assessment and bleeding risk assessment, which is helpful to further grade the risk of thrombosis to give corresponding thromboprophylactic measures [18]. At present, the Wells scale, which is widely used in clinical practice, is developed based on outpatient patients, so it is not applicable to inpatients with trauma [33]. The Padua scoring scale is applied to nonsurgical patients. At the same time, the Risk Assessment Profile for Thromboembolism (RAPT) can also be used for the risk assessment of VTE in trauma patients [34]. At present, the bleeding risk assessment models of VTE patients include the International Medical Prevention Registry on Venous Thromboembolism (IMPROVE), Kuijer score, and the European Multicenter Thromboembolism Registry (Registro Informatizado de la Enfermedad Thrombo-Embolica, RIETE) [35], among which the bleeding risk assessment model for VTE prevention only has the IMPROVE score, which is applicable to nonsurgical patients. The prediction ability of bleeding risk for surgical patients in VTE high-risk groups needs to be verified; however, other bleeding risk assessment models still lack validation and comparison of clinical use [36, 37]. The guidelines do not recommend any scoring model but list the risk factors related to bleeding [10, 35]. Medical staff can choose appropriate preventive measures according to the type of trauma, risk factors for VTE, and bleeding risk. The third piece of evidence describes the diagnostic evaluation of the diagnosis of VTE. DVT and pulmonary embolism are contraindications of IPC treatment and should be identified early in the prevention process. When DVT or pulmonary embolism is suspected, laboratory examination and imaging examination are required to make a definite diagnosis regardless of whether the clinical manifestation is typical.

### 4.2. Contraindications and Applicable Conditions

At present, routine VTE prevention methods include basic prevention, drug prevention, and mechanical prevention. The multimodal approach combining IPC and drug prevention is the most effective in reducing venous thromboembolism [38]. For patients with a moderate to high risk of VTE, if there is no contraindication for drug prevention, drug prevention is the first choice for VTE prevention, and drug prevention combined with mechanical prevention has a lower incidence rate of VTE than drug prevention alone. If there are contraindications, mechanical prevention can be an important choice. For low-risk patients, mechanical prevention can also effectively reduce the incidence rate of VTE [39]. Domestic and foreign guidelines recommend IPC as the first choice for mechanical prevention [25–27, 30]. Contraindications should be routinely screened before IPC treatment.

### 4.3. Treatment Strategy

1. At present, the effectiveness of IPC in preventing VTE has been recognized, but its compliance in clinical application is not high. Based on an observational study, the overall compliance with IPC treatment is only 29% [40, 41]. The possible reasons are that the patient or his family members do not know the purpose of use, the patient is intolerant or uncomfortable during use, and the use site may have adverse reactions (such as lower limb circulation disorder, skin allergy, or stress injury). Therefore, prior to IPC treatment, informed consent of the patient or family member should be obtained to explain the purpose of IPC treatment, precautions during treatment, and observation of adverse reactions. At the same time, limb evaluation and IPC device evaluation should be carried out to strengthen the key evaluation of the effectiveness of wearing the device, the patient's tolerance, comfort, and the skin of the use site. At present, there are various intermittent air pressure devices [31], but the guide does not make recommendations for specific IPC equipment [25]. In addition, there is no significant difference in the treatment effect between different inflating methods, and there is no significant difference in the treatment effect between different lengths of cuff [31]. When used, the appropriate IPC can be selected according to the wishes of the patient and the conditions of the hospital.
2. Traumatic patients have a tendency of thrombosis within 24 hours after injury, and their blood is also in a hypercoagulable state. Generally, this tendency is most obvious approximately 5 days after injury, and it begins to decline 14 days after injury. Therefore, if there is no contraindication, preventive measures should be started as soon as possible [42]. Generally, mechanical prevention is relatively safe. On the premise that the patient can tolerate it, the use time should be extended as much as possible, but the dynamic observation and evaluation of the patient and device in the treatment cannot be ignored. Medical staff should conduct dynamic observation and evaluation of patients and equipment during IPC treatment of patients. Once the possibility of new DVT or pulmonary embolism is suspected, the treatment should be stopped immediately, and the VTE early evaluation process should be entered. At present, the use of IPC in clinical treatment is arbitrary in the choice of time, and the length and frequency of use are also different. The lack of evidence-based support leads to certain blindness in treatment. In addition, there are differences in medical systems at home and abroad. Problems such as insufficient awareness and attention of domestic nursing staff on thrombosis prevention and lack of hospital equipment have led to large differences in the clinical use time of IPC [43]. Therefore, nursing staff should carefully select the use time of IPC by referring to high-quality evidence-based evidence and combining it with patients' own factors. During IPC treatment, due to the excessive pressure on the local part of the limb, arterial blood could not reach the extremities smoothly, resulting in limb ischemia and hypoxia, which could be manifested as pale skin, decreased skin temperature, limb numbness, intermittent claudication, etc. In order to prevent the occurrence of limb ischemia, the medical staff should fully evaluate whether the patient has any contraindication of IPC before operation and select the appropriate compression mode and duration according to the patient's condition. At the same time, the pressure injury related to IPC cannot be ignored. It is the local injury of skin and/or soft tissue caused by the compression of the air bag for a long time, which may lead to subcutaneous and soft tissue ischemia and necrosis, which can be manifested as skin redness, heat, pain, etc. In serious cases, there will be blister formation, skin ulcer, and even necrosis. During IPC treatment, if there is an undetected deep vein thrombosis falling off, it may cause PTE with dyspnea, chest pain, cough, hemoptysis, palpitation, and other main manifestations, which is the most serious complication. Before IPC treatment, the patient's condition should be fully understood, and the patient should be evaluated for no contraindications. If necessary, ultrasound screening of lower limb veins should be performed.

### 4.4. Training and Patient Education

In clinical practice, low compliance with IPC treatment is related not only to the above patient factors but also to medical staff factors. A lack of VTE-related training for medical staff will directly affect the effect of IPC mechanical prevention [44], and strengthening the training of medical staff is a technical guarantee to promote the implementation of VTE prevention measures. A current survey study shows that strengthening the training of medical staff can improve compliance with IPC treatment from 26% to 44% [38]. In addition, the information needs of patients are considered an important factor affecting compliance [38]. It is also important to carry out health education for patients or family members (long-term caregivers) receiving IPC treatment. The medical staff of the VTE high-risk department should communicate in a timely manner with the patients or their families (long-term caregivers), strengthen the popular science education of VTE prevention knowledge, and promote the implementation of VTE prevention measures.

## 5. Conclusion

VTE is one of the more common and preventable complications of trauma patients during hospitalization. As a strategy for thrombosis prevention, IPC is an important choice. This study uses scientific research methods to summarize the 27 best studies for IPC to prevent VTE in trauma patients, providing an evidence-based basis for the implementation of clinical IPC. We also want to see the update of IPC equipment. Wall et al. [45] used a new portable mechanical device (Wearable Intermittent Compression, WIC) for intermittent and continuous compression of leg grading to replace the traditional IPC mechanical system, making the compression device work in a mode similar to IPC but with shorter and faster cycle. The study showed that the new device significantly increased the venous flow compared with the baseline treatment of ECS and the traditional IPC treatment. In addition, WIC solves the additional limitations of IPC treatment by providing more flexible material selection, including breathable fabrics and less overall contact surface area. Mosti and Partsch [46] designed a new portable battery-driven compression device, which can automatically adjust the pressure to adapt to the change of body position and connect to the wrapped leg sets with different material hardness. In order to standardize the assessment, prevention, and treatment of VTE in trauma patients, we will record the leg ejection fraction (EF) and apply compression pressure under different pressure materials when lying, standing, and walking. In addition, in the process of promoting the implementation of evidence, it is also necessary to note that this study only includes the published literature. It is recommended that medical personnel should fully and comprehensively evaluate the clinical situation in the process of clinical application of evidence and apply the best evidence in combination with the wishes of patients and the conditions of hospitals to improve the effectiveness of IPC in preventing thrombosis. Second, although there are few reports of adverse events related to IPC treatment, the safety of IPC application should also be considered by medical staff. In the selection of IPC, medical staff should select the appropriate IPC according to the actual situation of patients to ensure its safety. In addition, compliance with the clinical application of IPC is relatively low, so we should start from two aspects before the application of evidence. We should not only increase the number of IPCs but also strengthen the training of VTE prevention knowledge and practical skills of medical staff to provide good health education for patients and their families to improve compliance with the clinical application of IPC.

## Figures and Tables

**Table 1:** General characteristics of the 19 included studies.

Number	Literature	Publication year	Source	Nature	Subject
1	Nathalie and Vicky	2021	Best practice	Guideline	Venous thromboembolism prophylaxis
2	Elizabeth	2020	UpToDate	Clinical decision	Thromboembolism and prevention in the severely injured trauma patient
3	JBI	2021	JBI	Summary of evidence	Orthopedic surgery: mechanical prophylaxis for venous thromboembolism
4	Hillegass	2016	UpToDate	Guideline	Role of physical therapists in the management of individuals at risk for diagnosed with venous thromboembolism: evidence-based clinical practice guideline
5	Kakkos	2021	UpToDate	Guideline	European Society for Vascular Surgery (ESVS) 2021 Clinical Practice Guidelines on the management of venous thrombosis
6	Anderson	2019	UpToDate	Guideline	American Society of Hematology 2019 guidelines for management of venous thromboembolism: prevention of venous thromboembolism in surgical hospitalized patients
7	NICE	2019	NICE	Guideline	Venous thromboembolism in over 16 years: reducing the risk of hospital-acquired deep vein thrombosis or pulmonary embolism
8	AHRQ	2015	AHRQ	Guideline	Preventing hospital-associated venous thromboembolism: a guide for effective quality improvement, 2^nd^ ed
9	SIGN	2014	SIGN	Guideline	Prevention and management of venous thromboembolism
10	Kearon	2012	PubMed	Guideline	Antithrombotic therapy for VTE disease: antithrombotic therapy and prevention of thrombosis, 9^th^ ed: American College of Chest Physicians Evidence-Based Clinical Practice Guidelines
11	Kahn	2012	PubMed	Guideline	Prevention of VTE in nonsurgical patients: antithrombotic therapy and prevention of thrombosis, 9^th^ ed: American College of Chest Physicians Evidence-Based Clinical Practice Guidelines
12	Falck-Ytter	2012	PubMed	Guideline	Prevention of VTE in orthopedic surgery patients: antithrombotic therapy and prevention of thrombosis, 9^th^ ed: American College of Chest Physicians Evidence-Based Clinical Practice Guidelines
13	Spahn	2019	PubMed	Guideline	The European guideline on management of major bleeding and coagulopathy following trauma: fifth edition
14	Liew	2017	PubMed	Guideline	Asian venous thromboembolism guidelines: updated recommendations for the prevention of venous thromboembolism
15	Rappold	2021	PubMed	Expert consensus	Venous thromboembolism prophylaxis in the trauma intensive care unit: an American Association for the Surgery of Trauma Critical Care Committee Clinical Consensus Document
16	Lozano	2013	Cochrane Library	Systematic review	Thromboprophylaxis for trauma patients
17	Ibrahim	2015	PubMed	Systematic review	Effect of compression devices on preventing deep vein thrombosis among adult trauma patients: a systematic review
18	Pavon	2016	PubMed	Systematic review	Effectiveness of intermittent pneumatic compression devices for venous thromboembolism prophylaxis in high-risk surgical patients: a systematic review
19	O'Connell	2016	PubMed	Systematic review	The use of intermittent pneumatic compression in orthopedic and neurosurgical postoperative patients: a systematic review and meta-analysis

**Table 2:** Quality evaluation results of 12 guidelines.

Literature	Percentage of standardization in each field (%)						≥60% of fields (number)	≥30% of fields (number)	Recommended level (level)
	Scope and purpose	Participants	Strictness	Clarity	Applicability	Independence			
Nathalie and Vicky	97.22	58.33	64.58	80.56	89.58	95.83	5	6	*B*
Hillegass	91.67	86.11	84.38	91.67	87.50	83.33	6	6	*A*
Kakkos	94.44	91.67	90.63	97.22	81.25	87.50	6	6	*A*
Anderson	91.67	94.44	84.38	97.22	89.58	87.50	6	6	*A*
NICE	97.22	88.89	94.79	94.44	91.67	95.83	6	6	*A*
AHRQ	91.67	86.11	73.96	80.56	54.17	91.67	5	6	*B*
SIGN	97.22	86.11	58.33	97.22	81.25	95.83	5	6	*B*
Kearon	94.44	91.67	92.71	97.22	91.67	95.83	6	6	*A*
Kahn	91.67	94.44	92.71	97.22	93.75	95.83	6	6	*A*
Falck-Ytter	97.22	91.67	92.71	97.22	91.67	95.83	6	6	*A*
Spahn	91.67	94.44	92.71	97.22	91.67	95.83	6	6	*A*
Liew	80.56	80.56	54.17	66.67	54.17	87.50	4	6	*B*

**Table 3:** Summary of evidence for the prevention of venous thromboembolism in trauma patients with intermittent pneumatic compression devices.

Project	Summary of evidence	Evidence level	Recommended level
*Pretreatment evaluation*			
Risk assessment	(1) The risk factors for VTE in trauma patients include spinal cord injury, head injury, lower limb fracture, pelvic fracture, need for surgical intervention, age ≥40 years old, femoral vein intubation, venous injury, ventilator use days >3 days, long-term braking, extended hospital stay, and high injury severity score.	4a	*B*
	(2) Assessment content of bleeding risk. (1) Patient factors: age, body mass, liver and kidney function, coagulation function, etc. (2) Primary diseases. (3) Complicated diseases (uncontrolled hypertension, active bleeding, history of massive hemorrhage disease, etc.). (4) Combined medication (antiplatelet drugs, anticoagulant drugs or thrombolytic drugs, etc.). (5) Whether there is invasive operation or surgery.	5b	*B*
	(3) VTE risk assessment and bleeding risk assessment should be carried out regularly for trauma patients, at least once every 48 hours; every time the clinical condition changes, re-evaluate at least every day. It is recommended that trauma patients use the Caprini scale or RAPT scale.	1b	*A*

Diagnostic assessment	(4) Before taking VTE preventive measures, for patients suspected of DVT or pulmonary embolism, the risk assessment scale of VTE can be used to make a preliminary diagnosis, and then the exclusion diagnosis can be made according to D-dimer. For patients at high risk of DVT, ultrasound examination is preferred. For patients with uncertain or infeasible ultrasound evaluation, computed tomography venography, magnetic resonance venography, or venography should be considered; for patients with high risk of pulmonary embolism, the diagnosis needs pulmonary artery imaging results (such as CT pulmonary angiography, magnetic resonance angiography, or digital subtraction angiography).	1c	*B*

*Contraindication and applicable conditions*			

Contraindication	(5) Contraindications should be routinely screened before application. (1) Severe leg edema or pulmonary edema caused by heart failure and congestive heart failure. (2) Suspect or confirm peripheral vascular disease. (3) Peripheral nerve or other sensory disorders. (4) Abnormal local conditions of lower extremities (such as dermatitis, gangrene, recent skin transplantation, open injury, and crush injury), severe arterial disease of lower extremities, other ischemic vascular diseases, and severe deformity of lower extremities. (5) Patients with deep venous thrombosis, thrombophlebitis, or pulmonary embolism should not receive IPC treatment. (6) Allergic to IPC device.	1a	*A*

Applicable conditions	(6) VTE risk is low. It is recommended to apply IPC for mechanical prevention.	2c	*A*
	(7) VTE risk is moderate, without high bleeding risk. It is recommended to use drug prevention or mechanical prevention, and drug prevention is preferred; for patients with high bleeding risk or extremely serious bleeding consequences, it is recommended to apply IPC for mechanical prevention.	2c	*A*
	(8) The risk of VTE is high, without high bleeding risk. It is recommended to recommend drug prevention combined with mechanical prevention; for patients with high bleeding risk or extremely serious bleeding consequences, it is recommended to apply IPC for mechanical prevention.	1b	*A*
	(1) For VTE high-risk trauma patients (such as surgical patients with acute spinal cord injury, brain injury, and spinal injury), drug prevention combined with IPC prevention is recommended if there is no contraindication, and IPC prevention is recommended if there is contraindication for drug prevention.	1b	*A*
	(2) For patients with high risk of bleeding (craniotomy, traumatic brain injury, spinal cord injury repair, major trauma, coagulation dysfunction, etc.) or patients with contraindications for drug thrombosis prevention (such as active hemorrhage and intracranial hemorrhage) or patients with hemodynamic instability caused by these conditions, drug prevention should be stopped and IPC mechanical prevention should be used.	2c	*A*
	(3) For patients who cannot move and are at risk of bleeding, it is recommended to use IPC for early mechanical thrombosis prevention.	1c	*A*
	(9) When the bleeding risk of patients with high risk of VTE is reduced, it is recommended to combine drugs and IPC for thrombus prevention within 24 hours after bleeding control until the patient can move.	1b	*B*
	(10) For patients who are undergoing lower limb amputation and contraindication of drug prevention or patients who are unable or unfit to take mechanical preventive measures on the affected side of the limb, consider IPC mechanical prevention on the opposite side of the leg at the time of admission, until the patient's mobility has improved.	1b	*B*

*Treatment strategy*			

Informed consent	(11) It is recommended to inform patients and their families in writing and obtain informed consent before applying IPC.	5b	*B*

Limb assessment	(12) Assess the skin hygiene, skin temperature, blood flow, dorsalis pedis artery pulsation, limb sensation, etc. of the lower limb every day, measure the leg circumference, and especially focus on the patients with decreased mobility or impaired skin integrity.	5b	*B*

Device evaluation	(13) Regularly check the IPC function status, patient comfort, performance, structural quality, battery-related functions (if equipped with batteries), and easy setting and other easy-to-use functions.	1b	*B*

Device selection	(14) Different types of IPC equipment differ in the number of air bags, compression cycle mode, inflation and deflation time, cuff pressure, cuff configuration, and portability. There are basically five different cuff configurations for lower limbs: foot compression, foot and calf compression, calf compression, calf and thigh compression, and whole limb compression.	1b	*B*
	(15) When selecting a specific IPC device, pay attention to the comfort and ease of use of the device, the acceptability, and cost of nursing staff and patients and select the IPC device according to the wishes of patients and the conditions of the hospital, which is helpful to select the appropriate IPC device.	1b	*B*

Timing	(16) For patients who have undergone skull, head and neck, spine, chest, and abdominal surgery, spinal cord injury, and severe trauma, intermittent inflating compression device shall be provided for 30 days or until the patient reaches his/her normal activity or discharge.	2b	*A*
	(17) Surgical patients usually begin IPC treatment during or after surgery and may require 10–14 days of thromboprophylaxis after surgery. For patients undergoing major orthopedic surgery, further extending the preventive measures to 35 days after surgery can further reduce the occurrence of DVT.	2b	*A*
	(18) For patients with moderate or higher risk of VTE during operation, IPC mechanical prevention is preferred, and it is recommended to start using before anesthesia until the patient can move normally or leave hospital.	5a	*B*
	(19) The IPC is recommended to ensure that the daily use time is at least 18 hours in the daytime and at night. For patients who are completely unable to move, the daily use time should be extended as much as possible under the premise of patient tolerance.	3d	*B*

Method	(20) Due to the different types, specifications, and manufacturers of IPC, there are also differences in the frequency, compression method, and compression strength of IPC. Please refer to the product manual for use.	5b	*B*
	(21) The patient needs to stay in bed during use, and the wrap of the cuff should start from the far end of the limb and gradually wind up.	5b	*B*
	(22) It is recommended to use IPC with lower limb sequential compression for VTE prevention in trauma patients.	3c	*B*

Security management	(23) During the treatment, the nurse observed the operation of the pressure pump, the power supply, the condition of the pipe and leg cover, and the machine alarm and kept communicating with the patient to understand their comfort and tolerance, to ensure the safety of the patient.	1b	*A*
	(24) In case of suspicious VTE signs during treatment, timely assessment and appropriate diagnostic evaluation shall be conducted to eliminate such potential fatal complications.	1b	*A*
	(25) Stop using IPC in the following cases: (1) the patient was suspected or confirmed to have DVT; (2) the patient was suspected or confirmed to have pulmonary embolism; (3) the patient has diagnosised pressure injury or severe lower limb circulation disorder; (4) the patient starts palliative treatment; (5) the patient's free movement; and (6) the patient has been discharged.	1c	*B*

*Training and patient education*			

Medical staff training	(26) It is suggested that medical staff should be fully trained to strengthen their VTE awareness, prevention awareness, and standardized management ability, to achieve timely assessment and treatment to prevent the occurrence of VTE.	5b	*B*

Health education for patients and their families	(27) We strongly encourage medical staff to carry out early mobilization for high-risk patients and provide adequate health education on VTE risks, signs, symptoms, and preventive measures for patients and their families, to reduce the possibility of thrombosis.	5b	*B*
